# Estimation of finite population mean using double sampling under probability proportional to size sampling in the presence of extreme values

**DOI:** 10.1016/j.heliyon.2023.e21418

**Published:** 2023-10-21

**Authors:** Jing Wang, Sohaib Ahmad, Muhammad Arslan, Showkat Ahmad Lone, A.H. Abd Ellah, Maha A. Aldahlan, Mohammed Elgarhy

**Affiliations:** a School of Economics and Management, Taiyuan Normal University, Jinzhong 030619, China; b Department of Statistics, Abdul Wali Khan University, Mardan, Pakistan; c Department of Mathematics and Statistics, Institute of Southern Punjab, Pakistan; d Department of Basic Sciences, College of Science and Theoretical Studies, Saudi Electronic University, Riyadh 11673, Saudi Arabia; e Mathematics Department, Faculty of Science, Al-Baha University, Saudi Arabia; f Department of Statisitcs, College of Science, University of Jeddah, Jeddah 23218, Saudi Arabia; g Mathematics and Computer Science Department, Faculty of Science, Beni-Suef University, Beni-Suef 62521, Egypt; h School of Finance and Economics, Jiangsu University, Zhenjiang 212013, China

**Keywords:** PPS, double sampling, Auxiliary variable, Bias, MSE, PRE

## Abstract

Values that are too large or small enough can be found in many data sets. Therefore, the estimator can yield ambiguous findings if several of the incredible deals are picked for the sample. When such extreme values occur, we propose improved estimators to determine the finite population means using double sampling based on probability proportional to size sampling (PPS). The properties of estimators are obtained up to the first order of approximations. When the size of the units varies widely, the PPS sampling technique may be employed. To determine the values of *Pi* when using PPS, we must be acquainted with the aggregate of the auxiliary variable $X_{i}$. However the designs and estimation techniques we have looked at so far are unsuccessful and are less effective when this information is difficult to locate or when other information is missing. The two-phase approach is preferable and more feasible in these kinds of circumstances. To demonstrate how effectively the recommended estimators performed, we used three actual data sets. We show mathematically and theoretically that the suggested estimators outperform alternative estimators.

## 1 Introduction

The effective use of auxiliary variables in survey sampling may boost the precision of estimators of the population parameter. The best statistical property estimates for population quantities, like mean, total, median, etc., are frequently searched for by researchers. For this, an illustrative sample of the population is needed. If the aggregate of concern is equivalent, choosing the entities can be done utilizing a SRS approach. It is necessary to know the aggregate constraints of the auxiliary variable in order to use the ratio, product, or regression methods of estimate. The ratio estimator plays an important role when there is a significant connection between the research and the auxiliary information. Apart from, the product estimator works effectively when there is a lack of association amongst the research and the auxiliary variable. By applicably adapting the auxiliary information, numerous researchers have developed various ratio estimators. Researchers can investigate this research by looking at [1] recommended on certail procedures of enlightening ratio and regression estimators [2]. recommended a better class of estimators for the mean of the population that use PPS sampling [3]. suggested that under linear transformation of the auxiliary variable, exponential estimators of the population mean be of the ratio type [4]. they reviewed a class of estimators of the population mean that hold satisfactorily against linear modification of the auxiliary information [5]. discussed a class of exponential ratio estimators consuming two auxiliary information [6]. studied mean estimate using quantile regression ratios under full and partial auxiliary information [7]. suggested robust quantile regression with two more variables for mean estimation [8]. recommended methods of enlightening estimators. The [9] discussed ameliorate estimation of mean using skewness and kurtosis of auxiliary character [10]. recommended a class of product estimators of population mean utilizing auxiliary information has been presented and questioned [11]. suggested an estimation of the population mean that was of the generalized exponential type and used auxiliary features [12]. recommended estimators of the mean of a population using simple random sampling that are based on robust ratios were proposed [13]. estimators for the mean of a population that make use of supplementary data and execute consecutive sampling on two occasions are recommended [14]. presented several imputation strategies for addressing missing information in two-sample consecutive sampling [15,16]. recommended estimation of population mean under probability proportional to size sampling with and without measurement errors.

In various situations, such as medical studies or surveys, it is common for the population sizes to diverge significantly. This can lead to variations in the probabilities or outcomes of different units within the population. For example, in a medical study examining a specific disease may be relatively small compared to the overall population size. This divergence in size can affect the probability of selecting individuals with the disease in a random sample. Researchers may need to account for this difference in population size and adjust their sampling methods or statistical analyses accordingly to ensure accurate representation and valid conclusions. Similarly, in surveys related to family income, the number of siblings within families can vary widely. This divergence in family size can influence the overall distribution of income levels within the survey population. It may be necessary to consider the different family sizes when analyzing the survey data or drawing conclusions about the relationship between family income and other variables. In situations like these, statistical techniques such as weighting, stratified random sampling, or other methods can be employed to address the divergent population sizes and account for the varying probabilities of units within the population. These techniques aim to provide accurate estimates and make valid inference despite the difference in population sizes. We utilize PPS sampling to deal with such an unequal probability. A PPS is an unequal random sampling in which, for each sampling component taken collectively, the chance of choices is proportional to an auxiliary variable. Let the context where we must evaluate the population in districts inside a province; we choose the auxiliary variable that has the determined relationship with the research variable.

For example.

(i) The aggregate of all districts inside the province (associated with research variable = 0.85).
(ii) The quantity of families in all societies inside the districts (association with the study variable = 0.98).

On the origin of these facts: (ii) more useful as an auxiliary variable.

Researchers can investigate this research by looking at [17] a discussion of using outliers to estimate the average of a population using a probability-based sampling design [18,19]. recommended PPS when outliers are present [20]. discussed combination of ratio and PPS estimators [21]. offered a more accurate estimation of the population size using PPS data [22]. discussed improved estimators in simple random sampling [23]. recommended on mixture of ratio and PPS estimators [24]. recommended substitute estimators in PPS sampling [25]. two auxiliary variables were suggested for improved estimate of the population mean using PPS.

Therefore when evidence like that is not readily accessible or when the auxiliary variable is not available, the earlier designs and estimating procedures do not produce capable results, and their efficiency decreases. Double-phase sampling is more beneficial and effective in this situation. The populations mean of the auxiliary information, which will be used in the evaluation or selection phase, can be estimated using an adequate initial sample.

For example:

On the condition of a single auxiliary information *X*, we take a sizeable investigative sample for estimating the population mean and only a subsample for computing the research variable *Y* because obtaining evidence on *X* is less expensive. This may imply allocating a portion of the assets to this large initial sample, resulting in a smaller sample size for computing the study variable. When the improvement in accuracy is significant compared to the rise in price due to the gathering of information on the auxiliary information for huge samples, this technique is favorable. The difficulty of calculating total buffalo milk production in a given region is an actual illustration of this situation. We use a community as the sampling element and the quantity of milk buffalo in a community as the auxiliary information in this study. Because the whole amount of milk buffalo in each community in the region may not be known, the investigator may choose a huge sample of communities and gather data on the number of milk buffalo in each village. This data is then utilized to calculate an estimate of *X*, the total number of milk buffalo in the area. The researchers are focused on an article regarding double-phase sampling at [26] who proposed the generalized regression estimator for two-phase tax record samples [27]. recommended the mean of a finite population can be estimated using linear regression and the ratio product [24]. presented double-sampling modified exponential estimators for the mean of a finite population [28]. recommended combining exponential functions for effective estimate when two-phase sampling is used [29]. in the context of stratified two-stage sampling, we talked about exponential chain ratio estimators [30]. consuming two auxiliary information in stratified two-phase sampling, a new, more accurate calibration estimator was presented [31]. recommended a family of estimators for predicting population mean from auxiliary proportions in single- and two-stage samples [32]. discussed a two-phase sampling method that uses a generalized methodology to estimate a finite population mean was suggested [33]. estimated the mean of a finite population using a mixed exponential-type estimator and a two-stage sampling design [34]. proposed that two-phase sampling could improve mean population estimates [35]. recommended an effective group of double-sampling estimators for the population mean [36]. for double-sampling the mean of a finite population, an exponential estimator of the chain-ratio type is proposed.

Our primary objectives are highlighted as follows.

1. In this paper, the primary objective of the contemporary effort is to estimate the finite population means using double sampling under PPS in the existence of extreme values (minimum and maximum values).
2. The numerical properties i.e. bias and MSE of the recommended estimator, are consequent up to the first order of approximation.
3. The application of the recommended estimator is highlighted through the use of real data sets from various domains.

## 2 Sampling methodology

Let a population Ψ = $\left\{\Psi_{1},\Psi_{2},\ldots ,\Psi_{N}\right\}$ of size *N* unlike elements. In the first phase, we draw an initial large sample of size “*m*” (*m* < *N*) from Ψ by making use of the SRSWOR sampling design and estimating the auxiliary information x. In the second phase, we take out a sub-sample of size “*n*” from the first phase of size “*m*”, i.e., (*n* < *m*) by SRSWOR or at first hand from Ψ, and notice both the study and auxiliary variables. Consider $y_{i},x_{i}$ and $z_{i}$ to be the study and auxiliary variables, respectively.

Let $P_{i}$ = $\frac{Z_{i}}{\sum_{i=1}^{N}Z_{i}}$, be the PPS to size for $i^{th}$ units, where

$${\bar{u}}_{11}=\frac{\sum_{i=1}^{n}u_{i11}}{n}={\bar{y}}_{pps},{\bar{v}}_{11}=\frac{\sum_{i=1}^{N}v_{i12}}{n}={\bar{x}}_{pps}\text{,}$$

$$\bar{Y}=\frac{1}{N}\sum_{i=1}^{N}y_{i},\text{and}\;\bar{X}=\frac{1}{N}\sum_{i=1}^{N}x_{i}\text{,}$$

$$u_{i11}=\frac{1}{NP_{i}}\;y_{i},v_{i12}=\frac{1}{NP_{i}}\;x_{i},s_{u11}^{2}=\sum_{i=1}^{N}P_{i}{\left(u_{i11}-\bar{Y}\right)}^{2},s_{v12}^{2}=\sum_{i=1}^{N}P_{i}{\left(v_{i12}-\bar{X}\right)}^{2}\text{,}$$

$$\rho_{uv}=\frac{\sum_{i=1}^{N}\left(u_{i11}-\bar{Y}\right)\left(v_{i12}-\bar{X}\right)}{s_{u11}s_{v12}},s_{u11}=\sqrt{P_{i}{\left(u_{i11}-\bar{Y}\right)}^{2}},s_{v12}=\sqrt{P_{i}{\left(v_{i12}-\bar{X}\right)}^{2}}\text{.}$$

Some real data sets include extreme values, e.g., when estimating the intelligence quotient (IQ), the brilliant students got (maximum) marks, and the weak students got (minimum) marks. If there are unexpectedly large or small elements in the population, the finite population mean is particularly delicate to unpredicted values. Furthermore, because the mean estimator is particularly delicate to such unpredicted findings, the population mean will either be ordinary or overstated depending on whether the sample contains large or small values. Consequently, if any of the surprising values are picked in the sample, the estimator can produce ambiguous conclusions. [37], suggested the following unbiased estimator to overcome this issue, which is given in equation (1).

(1) $${\hat{\bar{y}}}_{ss}=\left\{ \begin{array}{c} \bar{y}+s,\text{if}\;\text{the}\;\text{sample}\;\text{contains}\;\text{only}\;\text{minimum},\text{not}\;\text{maximum}\;\text{values}\\ \bar{y}-s,\text{if}\;\text{the}\;\text{sample}\;\text{contains}\;\text{only}\;\text{maximum},\text{not}\;\text{minimum}\;\text{values}\\ \bar{y,}\;\text{if}\;\text{sample}\;\text{contains}\;\text{all}\;\;\text{observation} \end{array}\right.$$

$$\text{Var}\left({\hat{\bar{y}}}_{ss}\right)=\varphi s_{u11}^{2}-\frac{2\varphi nc_{2}}{N-1}\left[\sigma_{u11}-nc_{1}\right]$$

The MSE of ${\hat{\bar{y}}}_{ss}$, at the unknown value of *c*, which is given in equation (2):

(2) $${\text{Var}\left({\hat{\bar{y}}}_{ss}\right)}_{\min}=\text{Var}\left(\bar{y}\right)\;‐\;\frac{\varphi {\sigma_{u11}}^{2}}{2\left(N-1\right)}\text{,}$$

where

$$\text{Var}\left(\bar{y}\right)=\varphi s_{u11}^{2}$$

[11]recommended population total under PPS, which are given in equation (3):

(3) $${\hat{\bar{y}}}_{pps}=\frac{1}{n}\sum_{i=1}^{n}\frac{\left(y_{i}+\varphi \right)}{p_{i}}\;‐\;N\;\varphi \text{,}$$

where, $p_{i}$ = $\frac{c_{xi+n}}{c_{xi+Nn}}$.

For estimation of the population means, we can also write equation (3) as given by:

$${\hat{\bar{y}}}_{pps}=\frac{1}{n}\sum_{i=1}^{n}\frac{\left(y_{i}+\varphi \right)}{{Np}_{i}}\;–\varphi$$

$\frac{\left(c_{xi+Nn}\right)}{Nn}\sum_{i=1}^{N}\frac{\left(y_{i}+\varphi \right)}{c_{xi+n}}$, when c = 1, n = 0, φ = 0

$${\hat{\bar{y}}}_{pps}=\frac{1}{n}\sum_{i=1}^{n}\frac{\left(y_{i}\right)}{{Np}_{i}}=\frac{1}{n}\sum_{i=1}^{n}u_{i11}={\bar{u}}_{11}={\bar{y}}_{pps}$$

The variance of ${\bar{y}}_{pps}$ is given in equation (4):

(4) $$\text{Var}\left({\hat{\bar{y}}}_{T,PPS}\right)=\varphi {\bar{u}}_{11}c_{u11}^{2}$$

The ratio and product estimators [38,39] which are given in equations (5), (6):

(5) $${\bar{y}}_{RT,PPS}={\bar{u}}_{11}\left(\frac{{\bar{X}}^{*}}{{\bar{v}}_{11}}\right)\text{,}$$

(6) $${\bar{y}}_{PT,PPS}={\bar{u}}_{11}\left(\frac{{\bar{v}}_{11}}{{\bar{X}}^{*}}\right)\text{,}$$

The MSE of ${\bar{y}}_{RT,PPS}$, and ${\bar{y}}_{PT,PPS}$ are given in equations (7), (8):

(7) $$\text{MSE}\left({\bar{y}}_{RT,PPS}\right)={\bar{Y}}^{2}\left\{\varphi c_{u11}^{2}+\varphi_{2}c_{v12}\left(c_{v12}-2\rho_{u11v12}c_{u11}\right)\right\}$$

and

(8) $$\text{MSE}\left({\bar{y}}_{PT,PPS}\right)={\bar{Y}}^{2}\left\{\varphi c_{u11}^{2}+\varphi_{2}c_{v12}\left(c_{v12}+2\rho_{u11v12}c_{u11}\right)\right\}$$

The regression estimator is given in equation (9):

(9) $${\bar{y}}_{RegT,PPS}={\bar{u}}_{11}+\beta \left({\bar{X}}^{*}-{\bar{v}}_{11}\right)\text{.}$$

The variance of regression is given in equation (10):

(10) $$\text{Var}\left({\bar{y}}_{RegT,PPS}\right)=\varphi s_{u11}^{2}+\varphi_{2}s_{u11}^{2}\left(1‐\;\rho_{u11v12}^{2}\right)\text{.}$$

Where.

φ = $\left(\frac{1}{n}-\frac{1}{N}\right)$ , $\varphi^{\prime }$ = $\left(\frac{1}{m}-\frac{1}{N}\right)$, $\varphi_{2}$ = $\left(\frac{1}{n}-\frac{1}{m}\right)$.

## 3 Suggested estimators

Some real data sets included extreme values, either very large or small. The efficiency of estimators may suffer in the manifestation of these extreme values. For example, while measuring the average export of goods, China may produce a large number of goods for the international market due to new technology and improved skills of its people, compared to Pakistan's small amount of goods due to poor management and lack of technology. Similarly, if we wish to know the average yearly wheat production in our country, we can see that wheat production in Punjab is extremely large as compared to other provinces. To deal with such an extreme values taking motivation from Refs. [17,18], we suggested an improved ratio, product, and regression type estimator for double phase with PPS sampling in the occurrence of extreme values. The recommended improved estimators are presented in three different situations.

Situation-I: Mean per unit estimator, given in equation (11)

(11) $${\bar{y}}_{T,PPS}=\left\{ \begin{array}{c} {\bar{u}}_{11}+c,\text{If}\;\text{the}\;\text{selected}\;\;\text{observation}\;\text{included}\;\text{small}\;\text{value}\;\text{of}\;u_{i11}\\ {\bar{u}}_{11}-c,\text{If}\;\text{the}\;\text{selected}\;\;\text{observation}\;\text{included}\;\text{large}\;\text{value}\;\text{of}\;u_{i11}\\ {\bar{u}}_{11},\text{If}\;\text{the}\;\text{selected}\;\;\text{observation}\;\text{included}\;\text{other}\;\text{values} \end{array}\right.$$

The optimal value of *C*, is given as:

$$C=\frac{\varphi \sigma_{u11}^{2}}{2\left(N-1\right)}\text{,}$$

The least variance at the value of *C* are given in equation (12):

(12) $$V\left({\hat{\bar{Y}}}_{T,PPS}\right)=V\left({\hat{\bar{y}}}_{T,PPS}\right)\;‐\;\frac{\varphi \sigma_{u11}^{2}}{2\left(N-1\right)}$$

Situation-II: When u and v are positively correlated.

When the correlation between u and v is positive, when the minimum cost of u is chosen, the collection of the minimum value of v is presumed. And for a maximum value of v, a maximum cost of u is assumed to be nominated. In such a scenario, we suggest the following improved ratio type estimator, which is given in equation (13).

(13) $${\hat{\bar{Y}}}_{RT,PPS}={\bar{u}}_{c11}\left(\frac{{\bar{X}}^{*}}{{\bar{v}}_{c21}}\right)\text{,}$$

or

$${\hat{\bar{Y}}}_{RT,PPS}=\left\{ \begin{array}{c} \left({\bar{u}}_{11}+c_{1}\right)\frac{\left({\bar{X}}^{*}+c_{2}\right)}{\left(\bar{v}+c_{2}\right)},\text{If}\;\text{the}\;\text{sample}\;\text{included}\;\text{small}\;\text{value}\;\text{of}\;u_{i11}\;\text{and}\;v_{i12}\\ \left({\bar{u}}_{11}-c_{1}\right)\frac{\left({\bar{X}}^{*}-c_{2}\right)}{\left(\bar{v}-c_{2}\right)},\text{If}\;\text{the}\;\text{sample}\;\text{included}\;\text{large}\;\text{value}\;\text{of}\;u_{i11}\;\text{and}\;v_{i12}\\ {\bar{u}}_{11}\left(\frac{{\bar{X}}^{*}}{\bar{v}}\right),\text{for}\;\text{all}\;\text{other}\;\text{samples} \end{array}\right.\text{,}$$

where $\left({\bar{u}}_{c11}={\bar{u}}_{11}+c_{1},{\bar{X}}_{c21}^{*}={\bar{X}}^{*}+c_{2},{\bar{v}}_{c21}={\bar{v}}_{11}+c_{2}\right)$. If the trial contains minimum values of *u* and *v*. $\left({\bar{u}}_{c11}={\bar{u}}_{11}-c_{1},{\bar{X}}_{c21}^{*}={\bar{X}}^{*}-c_{2},{\bar{v}}_{c21}={\bar{v}}_{11}-c_{2}\right)$. If a trial contains maximum values of *u* and *v*, and $\left({\bar{u}}_{c11}={\bar{u}}_{11},{\bar{X}}_{c21}^{*}={\bar{X}}^{*},{\bar{v}}_{c21}={\bar{v}}_{11}\right)$, for all further samples. Where $c_{1}$ and $c_{2}$ are sustained, its value y be decisive for optimal conditions.

The regression estimator is given in equation (14):

(14) $${\bar{y}}_{T,Reg1,PPS}={\bar{u}}_{c11}+b\;\left({\bar{X}}^{*}-{\bar{v}}_{c21}\right)\text{,}$$

where $\left({\bar{u}}_{c11}={\bar{u}}_{11}+c_{1},{\bar{v}}_{c21}={\bar{v}}_{11}+c_{2}\right)$ if the trial comprises u and v minimum. $\left({\bar{u}}_{c11}={\bar{u}}_{11}-c_{1},{\bar{v}}_{c21}={\bar{v}}_{11}-c_{2}\right)$ if the trial comprises u and v maximum, and $\left({\bar{u}}_{c11}={\bar{u}}_{11},{\bar{v}}_{c21}={\bar{v}}_{11}\right)$, for all other samples.

Situation-III: When u and v are negatively correlated.

While u and v are both negatively correlated with one another, the picking of a large assessment of *v* is expected to be accompanied by a small value of u. Similarly, when a small value of *v* is selected, it is expected to select a large value of *u*. Based on these situations, we suggested the following improved product type estimator, which is given in equation (15):

(15) $${\hat{\bar{Y}}}_{PT,PPS}={\bar{u}}_{c12}\left(\frac{{\bar{v}}_{c22}}{{\bar{X}}^{*}}\right)\text{,}$$

or

$${\hat{\bar{Y}}}_{PT,PPS}=\left\{ \begin{array}{c} \left({\bar{u}}_{11}+c_{1}\right)\frac{\left(\bar{v}+c_{2}\right)}{\left({\bar{X}}^{*}+c_{2}\right)},\text{If}\;\text{the}\;\text{sample}\;\text{included}\;\text{small}\;\text{value}\;\text{of}\;u_{i11}\;\text{and}\;\text{large}\;\text{values}\;\text{of}\;v_{i12}\\ \left({\bar{u}}_{11}-c_{1}\right)\frac{\left(\bar{v}-c_{2}\right)}{\left({\bar{X}}^{*}-c_{2}\right)},\text{If}\;\text{the}\;\text{sample}\;\text{included}\;\text{large}\;\text{value}\;\text{of}\;u_{i11}\;\text{and}\;\text{small}\;\text{values}\;\text{of}\;v_{i12}\\ \bar{u}\left(\frac{\bar{v}}{{\bar{X}}^{*}}\right),\text{for}\;\text{all}\;\text{other}\;\text{samples} \end{array}\right.$$

The regression estimator is given in equation (16):

(16) $${\bar{y}}_{T,Reg2,PPS}={\bar{u}}_{c12}+b\;\left({\bar{X}}^{*}-{\bar{v}}_{c22}\right)\text{,}$$

where $\left({\bar{u}}_{c12}={\bar{u}}_{11}+c_{1},{\bar{v}}_{c22}={\bar{v}}_{11}-c_{2}\right)$ if the sample comprises u and v minimum. $\left({\bar{u}}_{c12}={\bar{u}}_{11}-c_{1},{\bar{v}}_{c22}={\bar{v}}_{11}+c_{2}\right)$ if the sample comprises u and v maximum, and $\left({\bar{u}}_{c12}={\bar{u}}_{11},{\bar{v}}_{c22}={\bar{v}}_{11}\right)$, for all other samples.

To find out biases and MSE we explain the relative error term and their expectation given as:

Let

$$\epsilon_{0}=\frac{{\bar{u}}_{11}-\bar{U}}{\bar{U}},\epsilon_{1}=\frac{{\bar{v}}_{11}-\bar{V}}{\bar{V}},{\epsilon_{1}}^{\prime }=\frac{{\bar{X}}^{*}-\bar{V}}{\bar{V}}\text{,}$$

$$E\;\left(\epsilon_{0}\right)=E\;\left(\epsilon_{1}\right)=E\;\left({\epsilon_{1}}^{\prime }\right)=0\text{.}$$

$$E\;\left({\epsilon_{0}}^{2}\right)=\left[\frac{\varphi }{{\bar{Y}}^{2}}\left(s_{u11}^{2}-\frac{2nc_{2}}{N-1}\left[\sigma_{u11}-nc_{1}\right]\right),E\left({\epsilon_{1}}^{2}\right)=\frac{\varphi }{{\bar{X}}^{2}}\;\left(s_{v12}^{2}-\frac{2mc_{2}}{N-1}\left[\sigma_{v12}-mc_{2}\right]\right)\right]\text{,}$$

$$E\;\left({\epsilon_{1}}^{\prime 2}\right)=\left[\frac{\varphi^{\prime }}{{\bar{X}}^{2}}\;\left(s_{v12}^{2}-\frac{2m^{\prime }c_{2}}{N-1}\left[\sigma_{v12}-mc_{2}\right]\right),E\left(\epsilon_{1}{\epsilon_{1}}^{\prime }\right)=\frac{\varphi^{\prime }}{{\bar{X}}^{2}}\;\left(s_{v12}^{2}-\frac{2m^{\prime }c_{2}}{N-1}\left[\sigma_{v12}-mc_{2}\right]\right)\right]\text{,}$$

$$E\;\left(\epsilon_{0}\epsilon_{1}\right)=\left[\frac{\varphi }{\bar{X}\bar{Y}}\left(s_{u11v12}-\frac{n}{N-1}\left[c_{2}\sigma_{u11}+c_{1}\sigma_{v}-{2nc}_{1}c_{2}\right]\right)\right]\text{,}$$

$$E\;\left(\epsilon_{0}{\epsilon_{1}}^{\prime }\right)=\left[\frac{\varphi^{\prime }}{\bar{X}\bar{Y}}\left(s_{u11v12}-\frac{m}{N-1}\left[c_{2}\sigma_{u11}+c_{1}\sigma_{v12}-{2mc}_{1}c_{2}\right]\right)\right]\text{,}$$

where

$$\sigma_{u}=u_{\max}\;‐\;u_{\min}\;\text{and}\;\sigma_{v}=v_{\max}‐\;v_{\min}\text{.}$$

By simplifying (12), in terms of e's.

${\hat{\bar{Y}}}_{RT,PPS}$ = $\bar{Y}\left(1+\epsilon_{0}\right){\left(1+\epsilon_{1}^{\prime }\right)}^{-1}$ ,or.

${\hat{\bar{Y}}}_{RT,PPS}$ = $\bar{Y}\left(\epsilon_{0}-\epsilon_{1}^{\prime }-\epsilon_{0}\epsilon_{1}^{\prime }+\epsilon_{1}^{\prime 2}\right)$.

Taking expectations from both sides, we have

$$\text{Bias}\;\left({\bar{y}}_{Tr,PPS}\right)=\bar{Y}\;\left(\varphi_{2}c_{v12}^{2}-\varphi_{2}c_{u11v12}\right)‐\frac{R}{\left(N-1\right)}\left\{\frac{2c_{2}}{\bar{X}}\left\{\left(n\varphi -m\varphi^{\prime }\right)\sigma_{v12}-c_{2}\left(n^{2}\varphi -m^{2}\varphi \right)\right\}-\left(n\varphi -m\varphi^{\prime }\right)\left(c_{2}\sigma_{u11}+c_{1}\sigma_{v12}\right)+2c_{1}c_{2}\left(n^{2}\varphi -m^{2}\varphi \right)\right\}\text{,}$$

where *R* = $\frac{\bar{Y}}{\bar{X}}$.

Unique values of $c_{1}$ and $c_{2}$ are not possible, because we have one equation and two unknown values.

$$c_{1\left(optimal\right)}=\frac{\sigma_{u11}}{\sigma_{v12}}\text{,}$$

$$c_{2\left(optimal\right)}=\left\{\frac{\left(N-1\right)\sigma_{u11}-mR\sigma_{v12}}{2mR\left(N-m-n\right)}\right\}$$

Putting the ideal values of $c_{1\left(optimal\right)}$ and $c_{2\left(optimal\right)}$, the least MSE of ${\hat{\bar{Y}}}_{RT,PPS}$, is given in equation (17):

(17) $$\text{MSE}\;\left({\hat{\bar{Y}}}_{RT,PPS}\right)=\text{MSE}\;\left({\bar{y}}_{Tr,PPS}\right)\;‐\;\frac{1}{2mN\left(N-1\right)}\left[\begin{array}{c} \sigma_{v}\left\{\left(N-n\right)\sigma_{u11}+2R\left(n-m\right)\sigma_{v12}\right\}\\ +\frac{\left(n-m\right){\left\{\left(N-n\right)\sigma_{u11}-mR\sigma_{v12}\right\}}^{2}}{m\left(N-m-n\right)} \end{array}\right]$$

Similarly, the bias of product type estimator is given:

B $\left({\hat{\bar{Y}}}_{PT,PPS}\right)$ = $\left[\frac{\varphi }{\bar{X}\bar{Y}}\left\{s_{u11v12}-\frac{n}{N-1}\left[c_{2}\sigma_{u11}+c_{1}\sigma_{v12}-{2nc}_{1}c_{2}\right]\right\}\right]$.

The MSE of product type estimator is given in equation (18):

(18) $$\text{MSE}\;\left({\hat{\bar{Y}}}_{PT,PPS}\right)=\text{MSE}\;\left({\bar{y}}_{PT,PPS}\right)\;‐\;\frac{1}{2mN\left(N-1\right)}\left[\begin{array}{c} \sigma_{v12}\left\{\left(N-n\right)\sigma_{u11}+2R\left(n-m\right)\sigma_{v12}\right\}\\ +\frac{\left(n-m\right){\left\{\left(N-n\right)\sigma_{u11}-mR\sigma_{v12}\right\}}^{2}}{m\left(N-m-n\right)} \end{array}\right]$$

In circumstance of positive correlation, the variance of ${\bar{y}}_{T,Reg1,PPS}$, given in equation (19);

(19) $$\text{Var}\;\left({\bar{y}}_{T,Reg1,PPS}\right)=\text{MSE}\;\left({\bar{y}}_{RegT,PPS}\right)‐\;\frac{1}{2mN\left(N-1\right)}\left[\begin{array}{c} \sigma_{v12}\left\{\left(N-n\right)\sigma_{u11}-2\beta \left(n-m\right)\sigma_{v12}\right\}\\ +\frac{\left(n-m\right){\left\{\left(N-n\right)\sigma_{u11}-mR\sigma_{v12}\right\}}^{2}}{m\left(N-m-n\right)} \end{array}\right]$$

In circumstance of negative correlation, the variance of ${\bar{y}}_{T,Reg2,PPS}$, given in equation (20):

(20) $$\text{Var}\;\left({\bar{y}}_{T,Reg2,PPS}\right)=\text{MSE}\;\left({\bar{y}}_{RegT,PPS}\right)\;‐\;\frac{1}{2mN\left(N-1\right)}\left[\begin{array}{c} \sigma_{v12}\left\{\left(N-n\right)\sigma_{u11}+2\beta \left(n-m\right)\sigma_{v12}\right\}\\ +\frac{\left(n-m\right){\left\{\left(N-n\right)\sigma_{u11}-mR\sigma_{v12}\right\}}^{2}}{m\left(N-m-n\right)} \end{array}\right]$$

Generally, we can write the variance of the regression estimator as given in equation (21):

(21) $$\text{Var}\;\left({\hat{\bar{Y}}}_{RegGT,PPS}\right)=\text{MSE}\;\left({\bar{y}}_{RegT,PPS}\right)\;‐\;\frac{1}{2mN\left(N-1\right)}\left[\begin{array}{c} \sigma_{v12}\left\{\left(N-n\right)\sigma_{u11}+2\;\left|\beta \right|\left(n-m\right)\sigma_{v12}\right\}\\ +\frac{\left(n-m\right){\left\{\left(N-n\right)\sigma_{u11}-mR\sigma_{v12}\right\}}^{2}}{m\left(N-m-n\right)} \end{array}\right]$$

## 4 Efficiency comparison

In this section, we equate theoretically the suggested estimators with existing counterparts.

(i) By taking (4) and (12)

Var(${\hat{\bar{Y}}}_{T,PPS}$) < Var(${\hat{\bar{y}}}_{T,PPS}$), or

Var(${\hat{\bar{y}}}_{T,PPS}$) - Var(${\hat{\bar{Y}}}_{T,PPS}$) > 0

$\frac{\varphi \sigma_{u11}^{2}}{2\left(N-1\right)}>$ 0

(ii) By taking (7) and (17)

MSE $\left({\hat{\bar{Y}}}_{RT,PPS}\right)$ < MSE(${\bar{y}}_{RT,PPS}$), or

MSE(${\bar{y}}_{RT,PPS}$) - MSE $\left({\hat{\bar{Y}}}_{RT,PPS}\right)$ > 0

$\frac{1}{2mN\left(N-1\right)}\left[\begin{array}{c} \sigma_{v12}\left\{\left(N-n\right)\sigma_{u11}+2R\left(n-m\right)\sigma_{v12}\right\}\\ +\frac{\left(n-m\right){\left\{\left(N-n\right)\sigma_{u11}-mR\sigma_{v12}\right\}}^{2}}{m\left(N-m-n\right)} \end{array}\right]>$ 0

(iii) By taking (8) and (18)

MSE $\left({\hat{\bar{Y}}}_{PT,PPS}\right)$ < MSE(${\bar{y}}_{PT,PPS}$), or

MSE(${\bar{y}}_{PT,PPS}$) - MSE $\left({\hat{\bar{Y}}}_{PT,PPS}\right)$ > 0

$\frac{1}{2mN\left(N-1\right)}\left[\begin{array}{c} \sigma_{v12}\left\{\left(N-n\right)\sigma_{u11}+2R\left(n-m\right)\sigma_{v12}\right\}\\ +\frac{\left(n-m\right){\left\{\left(N-n\right)\sigma_{u11}-mR\sigma_{v12}\right\}}^{2}}{m\left(N-m-n\right)} \end{array}\right]>$ 0

(iv) By taking (10) and (19)

Var $\left({\bar{y}}_{T,Reg1,PPS}\right)$ < Var(${\bar{y}}_{RegT,PPS}$), or

Var(${\bar{y}}_{RegT,PPS}$) - Var $\left({\bar{y}}_{T,Reg1,PPS}\right)$ > 0

$\frac{1}{2mN\left(N-1\right)}\left[\begin{array}{c} \sigma_{v12}\left\{\left(N-n\right)\sigma_{u11}-2\beta \left(n-m\right)\sigma_{v12}\right\}\\ +\frac{\left(n-m\right){\left\{\left(N-n\right)\sigma_{u11}-mR\sigma_{v12}\right\}}^{2}}{m\left(N-m-n\right)} \end{array}\right]$> 0

(v) By taking (10) and (20)

Var $\left({\bar{y}}_{T,Reg2,PPS}\right)$ < Var(${\bar{y}}_{RegT,PPS}$), or

Var(${\bar{y}}_{RegT,PPS}$) - Var $\left({\bar{y}}_{T,Reg2,PPS}\right)$ > 0

$\frac{1}{2mN\left(N-1\right)}\left(\begin{array}{c} \sigma_{v}\left\{\left(N-n\right)\sigma_{u11}+2\beta \left(n-m\right)\sigma_{v12}\right\}\\ +\frac{\left(n-m\right){\left\{\left(N-n\right)\sigma_{u11}-mR\sigma_{v12}\right\}}^{2}}{m\left(N-m-n\right)} \end{array}\right)>$ 0

## 5 Numerical investigation

We took three data sets to determine the suggested estimator's efficiency with existing counterparts. The summary statistics of these data sets are given below:

**Data-I [Source:** [24]**]:**

*Y* = Expected fish caught throughout 1995,

*X* = expected fish caught throughout 1994,

Z = expected fish caught throughout 1993.

**Data-II [Source:** [24]**]:**

*Y* = Expected fish caught throughout 1995,

*X* = expected fish caught throughout 1993,

*Z* = expected number of fish caught throughout 1992.

**Data-III [Source:** [40] **]:**

*Y*=Output for 80 yard,

*X* = stable capital in a region,

*Z* = number of labors.

## 6 Discussion

**Fig. 1:**
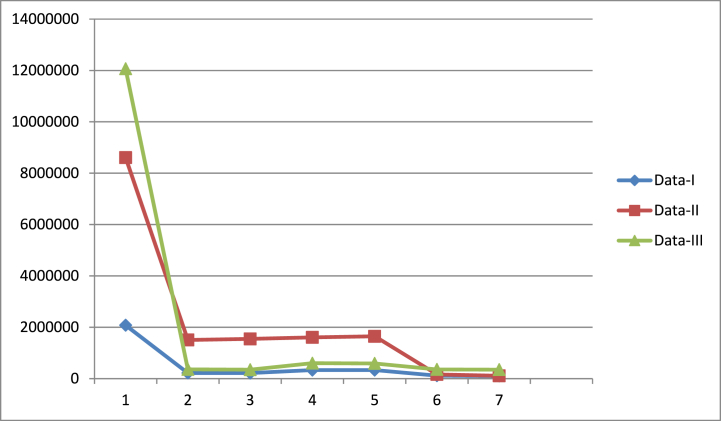
MSE of the suggested and existing estimators Fig. 1: On Y-axis, we put the values of mean square error, and on X-axis, we put the estimators.

**Fig. 2:**
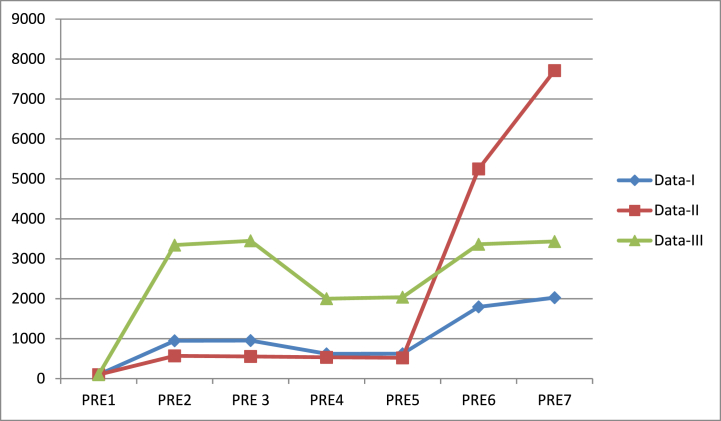
PRE of the suggested and existing estimators Fig. 2: On Y-axis, we put the values of mean square error, and on X-axis, we put the estimators.

As previously mentioned, we evaluated the performance of our suggested estimators using three real data sets. The proposed estimators are numerically and mathematically related to their current equivalents. The actual data are summarised statistically in Table 1, Table 2, Table 3. The MSE and PRE of our proposed and current counterparts are displayed in Table 4, Table 5. In phrase of MSE and PRE, it is detected that the suggested estimators are efficient than existing counterparts. The gain in data 2 is greater as compared to data 1 and data 3. Fig. 1 shows a comparison of estimators in terms of MSE. We plotted estimators on the *X*-axis and MSE values on the *Y*-axis. The estimator is more effective when you reduce the value of MSE. The efficiency of an estimator is directly related to the trend of lines. As the value of MSE is the minimum, the line graph shows the downward direction. Fig. 2 shows a comparison of estimators in terms of percentage relative estimators. When compared to their counterparts, our proposed estimators gain the most percentage relative efficiency. We plot estimators on the *X*-axis and values of PRE on the *Y*-axis. The higher the value of PRE, the better is the estimator. The trend line indicates an increasing path based on the PRE values.

**Table 1:** Summary statistic for Data-I.

*N* = 69	$\bar{X}$ = 4954.435	$c_{u11}$ = 0.4720461	$c_{v12}$ = 0.5049075	$u_{\max}$ = 11873.89	R = 0.9112843
*m* = 36	$\bar{Z}$ = 4591.072	$c_{u11}^{2}$ = 0.2228275	$c_{v12}^{2}$ = 0.2549316	$u_{\min}$ = 467.0002	$c_{u11v12}$ = 0.063411
$m_{1}$ = 20	$s_{u11}^{2}$ = 2420387	$s_{v12}^{2}$ = 2007238	$\rho_{u11v12}$ = 0.2660536	$v_{\max}$ = 18850.12	β = 0.292154
$\bar{Y}$ = 4514.899	$s_{u11}$ = 1555.759	$s_{v12}$ = 1416.77	$s_{u11v12}$ = 1,418,429	$v_{\min}$ = 894.5356

**Table 2:** Summary statistic for Data-II.

*N* = 69	$\bar{X}$ = 4591.072	$c_{u11}$ = 0.8523346	$c_{v12}$ = 1.509517	$u_{\max}$ = 20949.43	R = 0.983408
*m* = 36	$\bar{Z}$ = 4230.174	$c_{u11}^{2}$ = 0.7264743	$c_{v12}^{2}$ = 2.278641	$u_{\min}$ = 1002.527	$c_{u11v12}$ = 0.05634
$m_{1}$ = 20	$s_{u11}^{2}$ = 3,361,469	$s_{v12}^{2}$ = 2,245,140	$\rho_{u11v12}$ = 0.04379289	$v_{\max}$ = 54678.91	β = 0.05358
$\bar{Y}$ = 4514.899	$s_{u11}$ = 1833.488	$s_{v12}$ = 1498.379	$s_{u11v12}$ = 1,167,922	$v_{\min}$ = 1634.557

**Table 3:** Summary statistic for Data-III.

*N* = 80	$\bar{X}$ = 1126.463	$c_{u11}$ = 0.775310	$c_{v12}$ = 0.281887	$u_{\max}$ = 15586.8	R = 4.600808
*m* = 45	$\bar{Z}$ = 285.125	$c_{u11}^{2}$ = 0.6011057	$c_{v12}^{2}$ = 0.07946076	$u_{\min}$ = 2408.59	$c_{u11v12}$ = 0.12676
$m_{1}$ = 25	$s_{u11}^{2}$ = 10,568,817	$s_{v12}^{2}$ = 63257.12	$\rho_{u11v12}$ = 0.5800092	$v_{\max}$ = 1944.034	β = 7.497101
$\bar{Y}$ = 5182.637	$s_{u11}$ = 3250.972	$s_{v12}$ = 251.5097	$s_{u11v12}$ = 740038.3	$v_{\min}$ = 592.6127

**Table 4:** MSE of the existing and suggested estimators.

Estimators	Data-I MSE(.)	Data-II MSE(.)	Data-III MSE(.)
${\bar{y}}_{T,PPS}$	2,079,853	8,604,841	12,067,836
${\bar{y}}_{RT,PPS}$ ${\hat{\bar{Y}}}_{RT,PPS}$	219312.1 218,119	1,506,958 1,547,809	360886.7 349856.2
${\bar{y}}_{PT,PPS}$ ${\hat{\bar{Y}}}_{PT,PPS}$	334209.1 333016.1	1,609,051 1,649,902	603003.9 591973.5
${\bar{y}}_{RegT,PPS}$ ${\hat{\bar{Y}}}_{RegGT,PPS}$	115820.1 102615.2	163927.6 111587.4	358827.8 351424.7

**Table 5:** PRE of the existing and suggested estimators.

′Estimators	Data-I	Data-II	Data-III
${\bar{y}}_{T,PPS}$	100	100	100
${\bar{y}}_{RT,PPS}$ ${\hat{\bar{Y}}}_{RT,PPS}$	948.3534 953.5406	571.0073 555.9368	3343.941 3449.37
${\bar{y}}_{PT,PPS}$ ${\hat{\bar{Y}}}_{PT,PPS}$	622.3209 624.5504	534.7774 521.5365	2001.286 2038.577
${\bar{y}}_{RegT,PPS}$ ${\hat{\bar{Y}}}_{RegGT,PPS}$	1795.762 2026.846	5249.171 7711.299	3363.127 3433.975

## 7 Conclusion

In this paper, we have recommended an enhanced ratio, product, and regression type estimators for the estimation of finite population mean in double-phase with PPS sampling in the incidence of extreme values. The numerical expressions of properties are derived up to the first order of approximation. The purpose of this proposal is to enhance the accuracy and precision of mean estimation compared to existing estimators. To evaluate the efficiency of the recommended estimator, we conduct a comparative analysis with several existing counterparts. By comparing the performance of the proposed estimator against these alternatives, we aim to demonstrate its uniqueness and superiority. We used three actual data sets to obtain the MSEs and PRE. From the numerical results, recommended estimators perform well in terms of minimum mean square error and advanced PRE. It has been validated through empirical efficiency comparisons that our proposed estimators perform more effectively than the traditional estimators. The recommended estimators performed well, with the greatest gain in efficiency, and would perform well in applied surveys. The current work can be easily extended to yield an improved family of estimators under stratified random sampling and measurement error using the auxiliary information or attributes for estimation of population mean and variance. Additionally, it would be interesting to examine the efficiency of our recommended estimator in more complex survey settings, such as clustered and stratified sampling.

## Data availability

Data will be made available on request.

## Funding

There is no funding.

## CRediT authorship contribution statement

**Jing Wang:** Formal analysis. **Sohaib Ahmad:** Conceptualization, Investigation, Writing – original draft. **Muhammad Arslan:** Formal analysis. **Showkat Ahmad Lone:** Resources, Validation. **A.H. Abd Ellah:** Methodology, Validation. **Maha A. Aldahlan:** Conceptualization, Data curation. **Mohammed Elgarhy:** Data curation, Resources.

## Declaration of competing interest

The authors declare that they have no known cometing finincial intersts or personal relationships that could have appeared to influence the work reported in this paper.
